# Supplementary material for: Go/No-Go task engagement enhances population representation of target stimuli in primary auditory cortex
Source: Nat Commun. 2018 Jun 28;9:2529. doi: 10.1038/s41467-018-04839-9 (PMC6023878; doi:10.1038/s41467-018-04839-9)
Supplement: Supplementary file 1 — Supplementary Information [file 41467_2018_4839_MOESM1_ESM.pdf]

Go/No-Go task engagement enhances population representation of target stimuli in primary auditory cortex

Bagur et al

Supplementary Figures

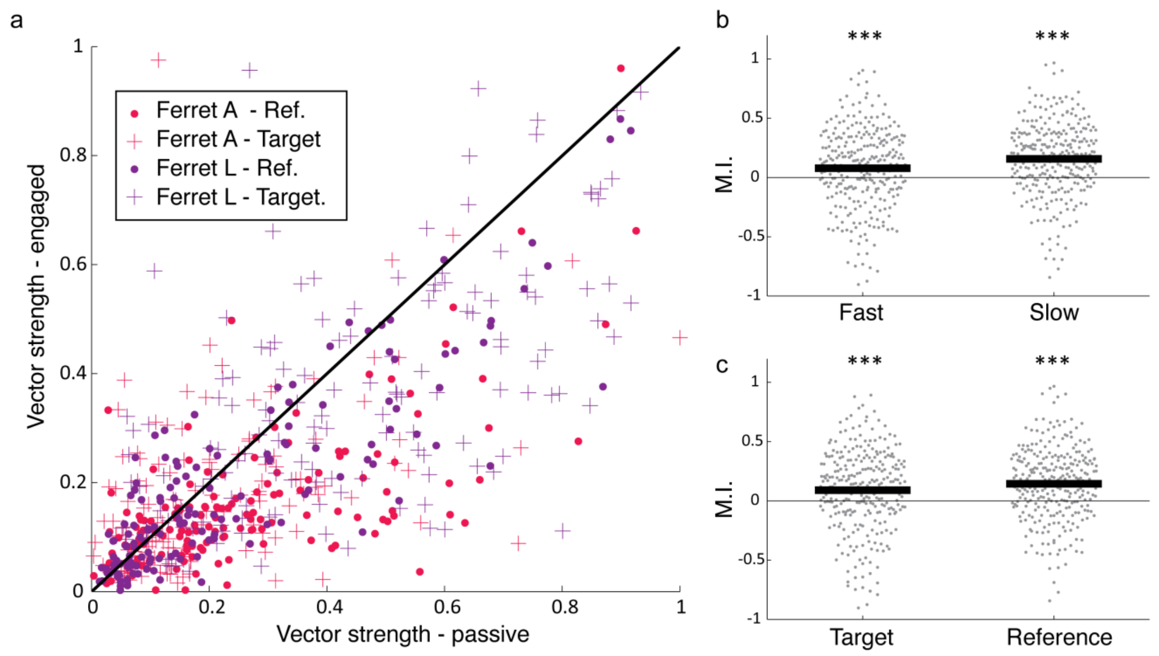

Supplementary Fig 1 Changes in stimulus entrainment between passive and engaged conditions

a. For each unit the vector strength for the reference and target click train is plotted in the engaged state vs the passive state. Animals are shown in different colors and stimuli with different markers. Note that most points are below the x=y line, showing higher phase locking in the passive state.

b. Modulation index of vector strength in task-engaged and passive states for fast and slow stimuli separately. (one-sample two-tailed Wilcoxon signed rank with mean 0, n=287; zval=-4.29, p=1.75e-5 & zval=-8.20, p=2.36e-16; ***: p<0.001).

c. Modulation index of vector strength in task-engaged and passive states for reference and target stimuli separately. (one-sample two-tailed Wilcoxon signed rank with mean 0, n=287; zval=-4.95, p=7.37e-7 & zval=-7.54, p=4.75e-14 ;***: p<0.001).

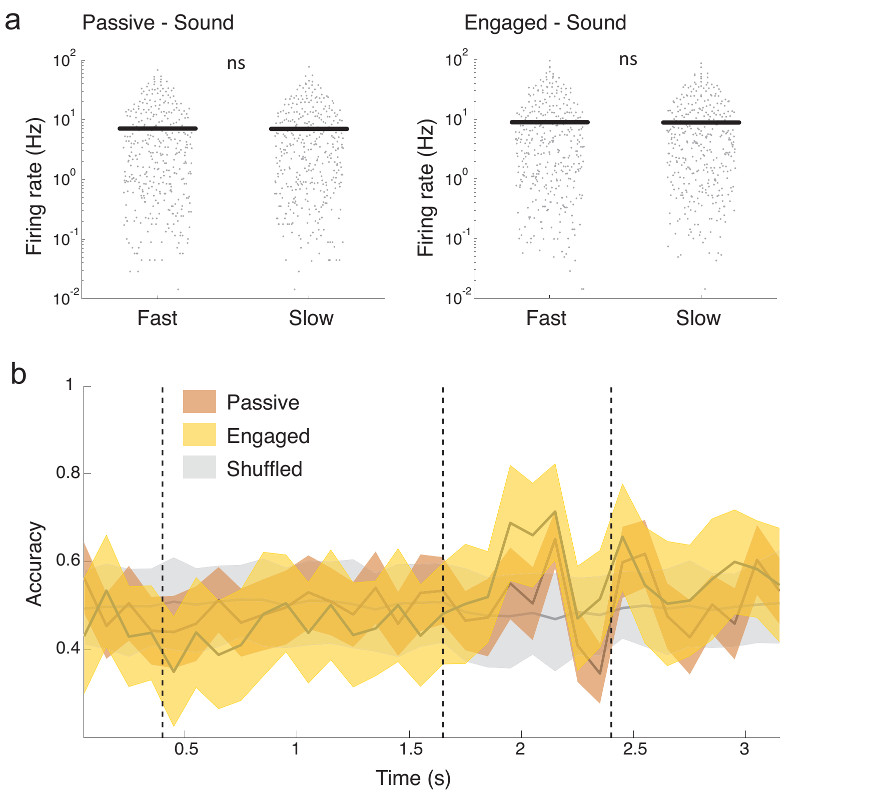

Supplementary Fig 2 Reference and target stimuli cannot be discriminated on the basis of population-averaged activity

a. Comparison of average firing rates on log scale in passive (left) and engaged (right) between fast and slow stimuli during the sound. (one-sample two-tailed Wilcoxon signed rank with mean 0, n=360; zval=-0.53, p=0.59 & zval=-0.25, p=0.8).

b. Accuracy of decoding in engaged and passive state using equal weights for all units. In grey, chance level performance evaluated on label-shuffled trials. Error bars are 1 std over 400 cross-validations

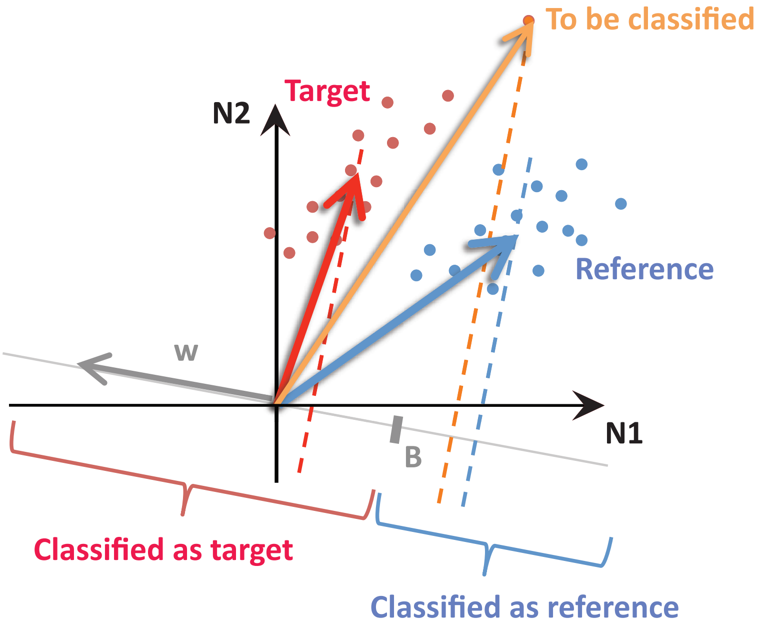

Supplementary Fig 3 Illustration of binary classifier
Illustration of binary classifier, see materials and methods.

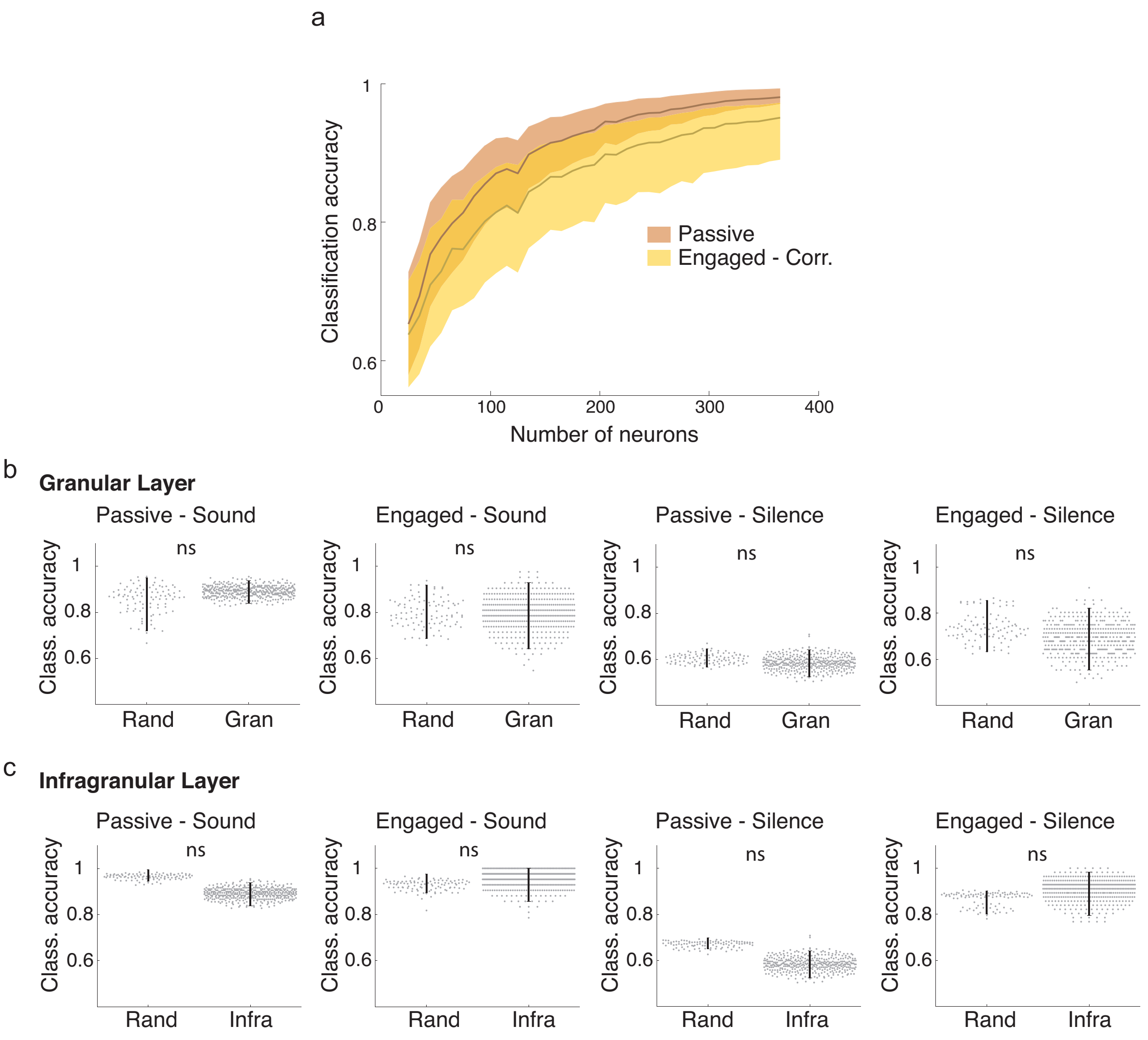

Supplementary Fig 4 Properties of the linear classifier

a. Effect on decoding accuracy of randomly adding units during the sound period. Error bar: 95% confidence intervals over 100 random selections of units.

b. Units taken from the granular layer only are used for classification and accuracy is compared with the same number (89) of randomly chosen units. Error bars: 95% confidence intervals. (100 sub-sampling procedures, 400 cross validations for accuracy using granular layer units; Bonferonni corrected p-value (8 tests): 0.0063; p=0.622, p=0.933, p=0.624, p=0.618)

c. Same as b but for infragranular layer (273 units). Error bars: 95% confidence intervals. (100 sub-sampling procedures, 400 cross validations for accuracy using granular layer units; Bonferonni corrected p-value (8 tests): 0.0063; p=0.0067, p=0.51, p=0.015, p=0.48)

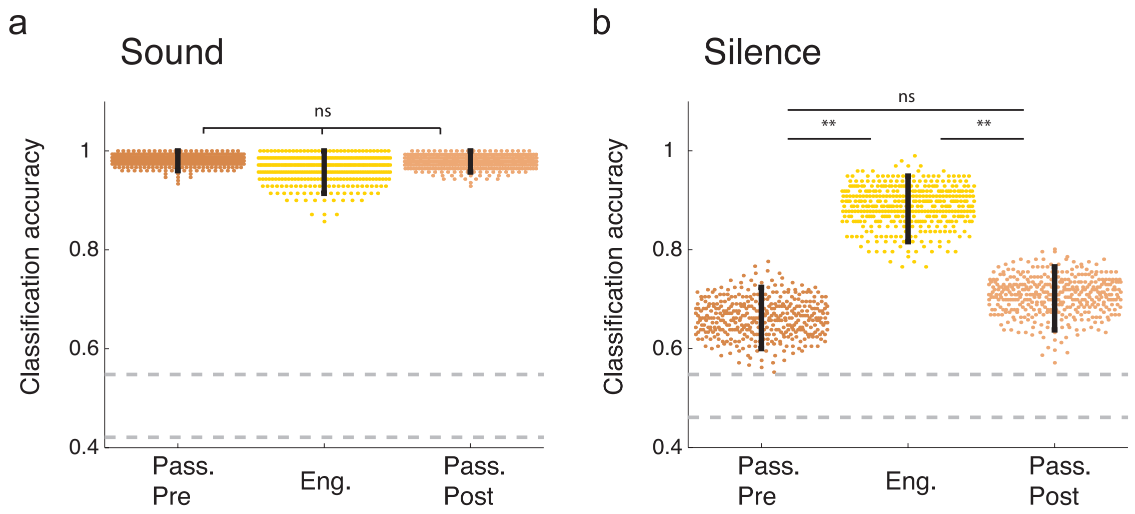

Supplementary Fig 5 Comparison of passive sessions before and after behavior

a. Comparison of accuracy during the sound period in the passive state before behavior, the task-engaged state and the passive state after behavior. Error bars represent 95% confidence intervals. (n=400 cross validations; pas.pre/eng: p=0.45, pas.pre/pas.post: p=0.74, eng/pas.post: p=0.58).

b. Comparison of accuracy during the silence period as in a. (n=400 cross validations; Bonferonni corrected p-value (3 tests): 0.0167; pas.pre/eng: p<0.0025, pas.pre/pas.post: p=0.43, eng/pas.post:, p<0.0025; **: p<0.01)

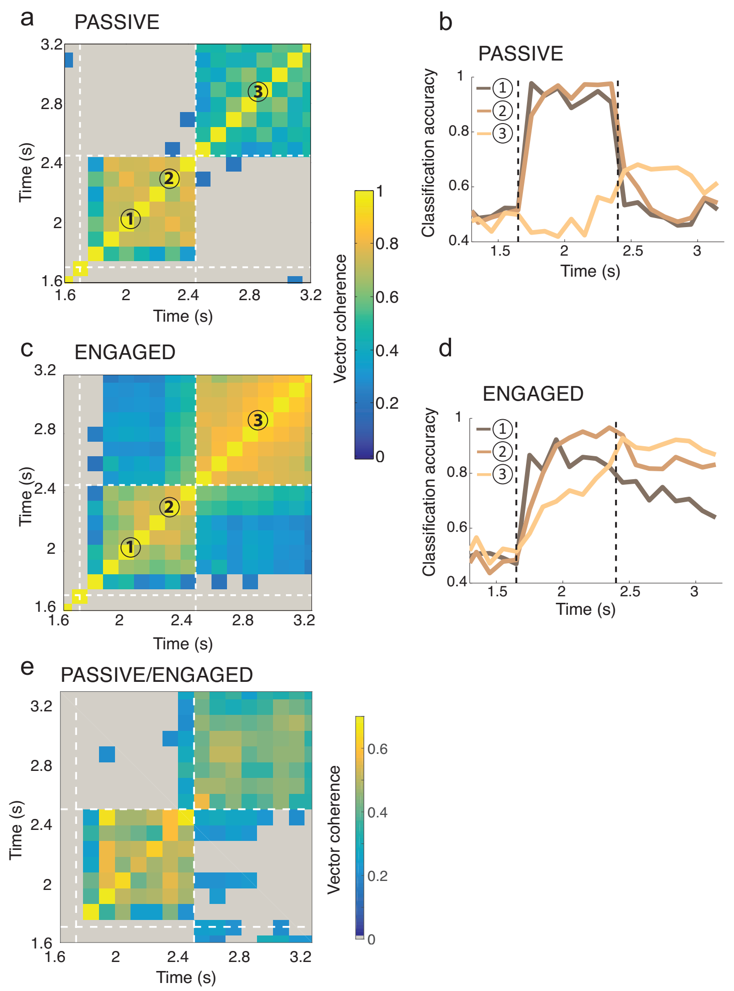

Supplementary Fig 6 Comparison of classifiers determined at different time-points and sessions

a. Classifier evolution in the passive state is shown in color as the correlation between decoding vectors at one time (y-axis) versus another (x-axis). Squares with below chance correlation values are shown in grey. Here, in the passive state, coding is homogeneous throughout the sound but does not allow for significant decoding in the silent period.

b. Decoding accuracy in the passive state using a decoder trained on the early (1) or late (2) sound or silence (3) periods. Accuracy is high throughout the sound for both early and late sound training but rapidly falls off during the silence. The decoder trained during the silence is only above chance after the sound has ended.

c. Classifier evolution in the task-engaged state as in (a). During the silence, coding is homogeneous.

d. As in (b) for the task-engaged state. The decoder trained during the early sound is specific to this period and performs poorly during the silence. Conversely, training late in the sound increases performance during the silence but decreases performance at the beginning of the sound. The accuracy of a decoder trained during the silence ramps up during sound presentation.

e. Correlation of passive and engaged decoding vectors throughout the trial. Vectors show stronger similarity during the sound than the silence between states. Note the different color scale, correlation between states is as expected lower than within states.

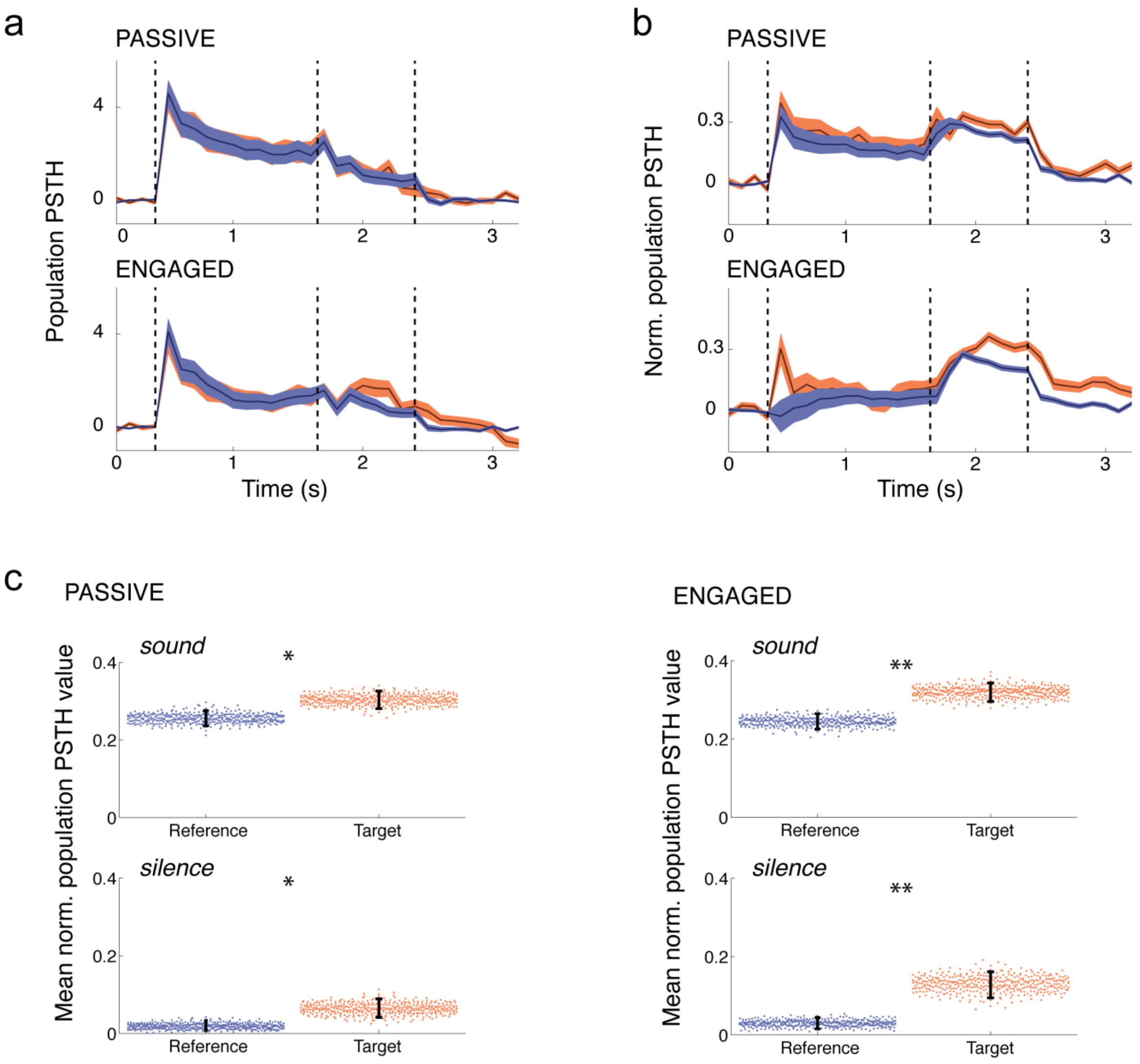

Supplementary Fig 7 Comparing population-averaged responses to target and reference stimuli

a. Average population PSTH on reference and target trials in the passive and task-engaged states. The PSTH of each neuron is baseline subtracted and then all PSTHs are averaged. Error bars: 95% C.I. after bootstrapping 400 times over all neurons (n=370).

b. Average normalized population PSTH on reference and target trials in the passive and task-engaged states. The PSTH of each neuron is baseline subtracted, corrected for the sign of its peak response to reference or target and normalized to its maximal response across states and stimuli. All normalized PSTHs are then averaged. Error bars: 95% C.I. after bootstrapping 400 times over all neurons (n=370).

c. Distance of reference and target from baseline after normalization as in (b). Results are shown for both states during the sound or the silence period. Error bars represent 95% confidence intervals. (n=400 cross validations; pass: $p=0.025$ & $p=0.025$, eng: $p<0.0025$ & $p<0.0025$;*: $p<0.05$; **: $p<0.01$)

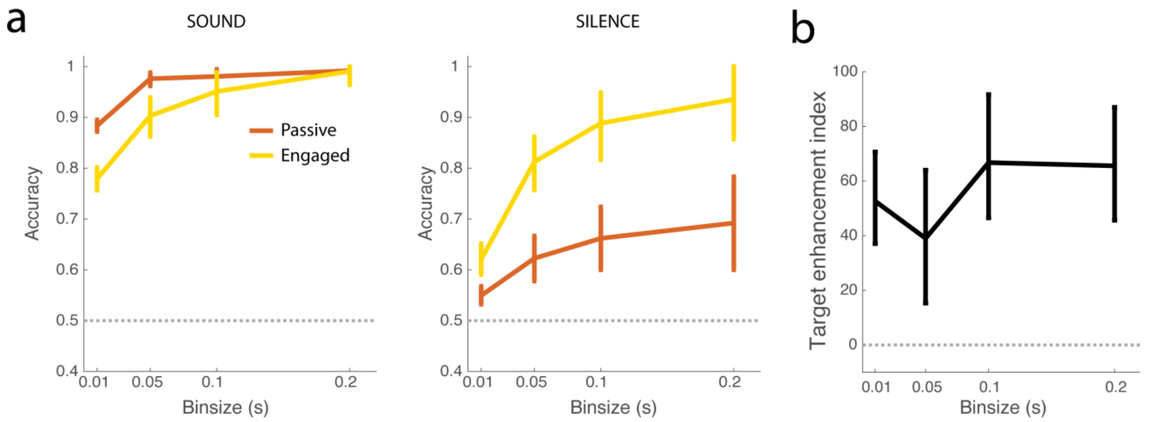

Supplementary Fig 8 Robustness of stimulus representation characteristics across a range of time scales

a. Accuracy of decoding during the sound (left) and silence (right) period in passive and engaged states calculated using a classifier determined with time bins of varying size. Error bars represent 95% confidence intervals. (n=400 cross validations)

b. Index of target enhancement by task engagement calculated during the sound period using a classifier determined with time bins of varying size. Note that for all time bins the value if significantly greater than 0, indicating a systematic enhancement of target driven encoding in the engaged state. Error bars represent 95% confidence intervals. (n=400 cross validations)

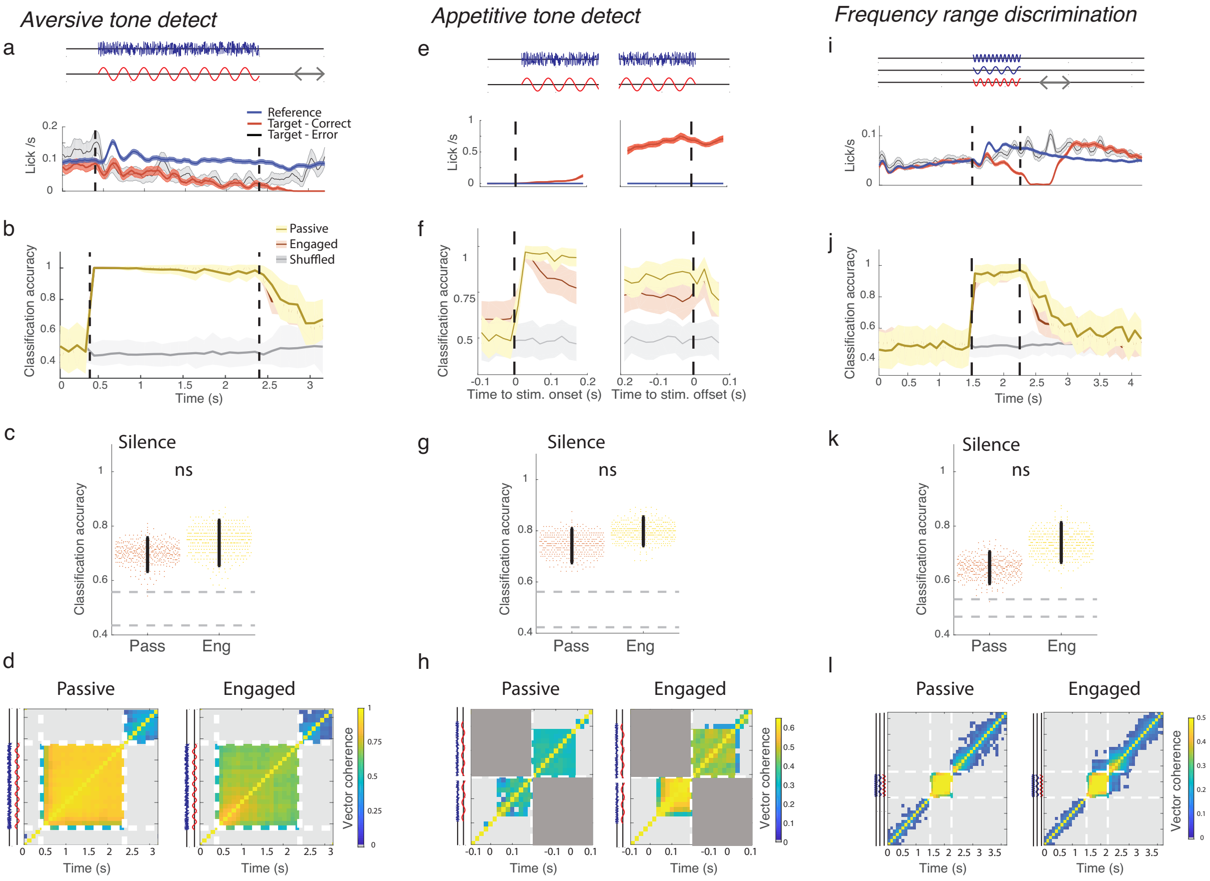

Supplementary Fig 9 Task structure and decoding of reference/target activity in a range of auditory go/no-go tasks

Three different tasks are considered: aversive tone detect (a-d), appetitive tone detect (e-h) and frequency range discrimination (i-l). Note that all analysis in this figure is done after excluding lick-responsive units for these tasks using the method described in Fig 4.

a, e, i. Top: Schematic of trial structure illustrating reference and target trials. Gray arrows show response window for the aversive tasks. Bottom: Licking frequency during correct target (red), reference (blue) and target error (gray) trials. Error bars are s.e.m over all trials.

b, f, j. Accuracy of stimulus classification in passive and engaged states. In grey, chance level performance evaluated on label-shuffled trials. Error bars represent 1 std calculated over 400 cross-validations.

c,g,k. Mean classifier accuracy during the post-sound silence period in passive and engaged conditions. Gray dotted lines give 95% confidence interval of shuffled trials. Error bars represent 95% confidence intervals. Note that accuracy is systematically above chance level in both conditions but does not change between the passive to the engaged state. (n=400 cross validations; p=0.21,0.18,0.055)

d,h,l. Classifier evolution in the passive (left) and engaged (right) state is shown in color as the correlation between decoding vectors at one time (y-axis) versus another (x-axis). Squares with below chance correlation values are shown in grey. For the appetitive tone detect task the overlap between sound onset and sound offset periods is not calculated as the difference in trial durations causes different overlaps in time on a trial to trial basis between the two. Note that the sound and silence periods in all tasks rely on different decoding vectors and in the case of the frequency range discrimination task, there is a progressive shift in the engaged state between decoders.

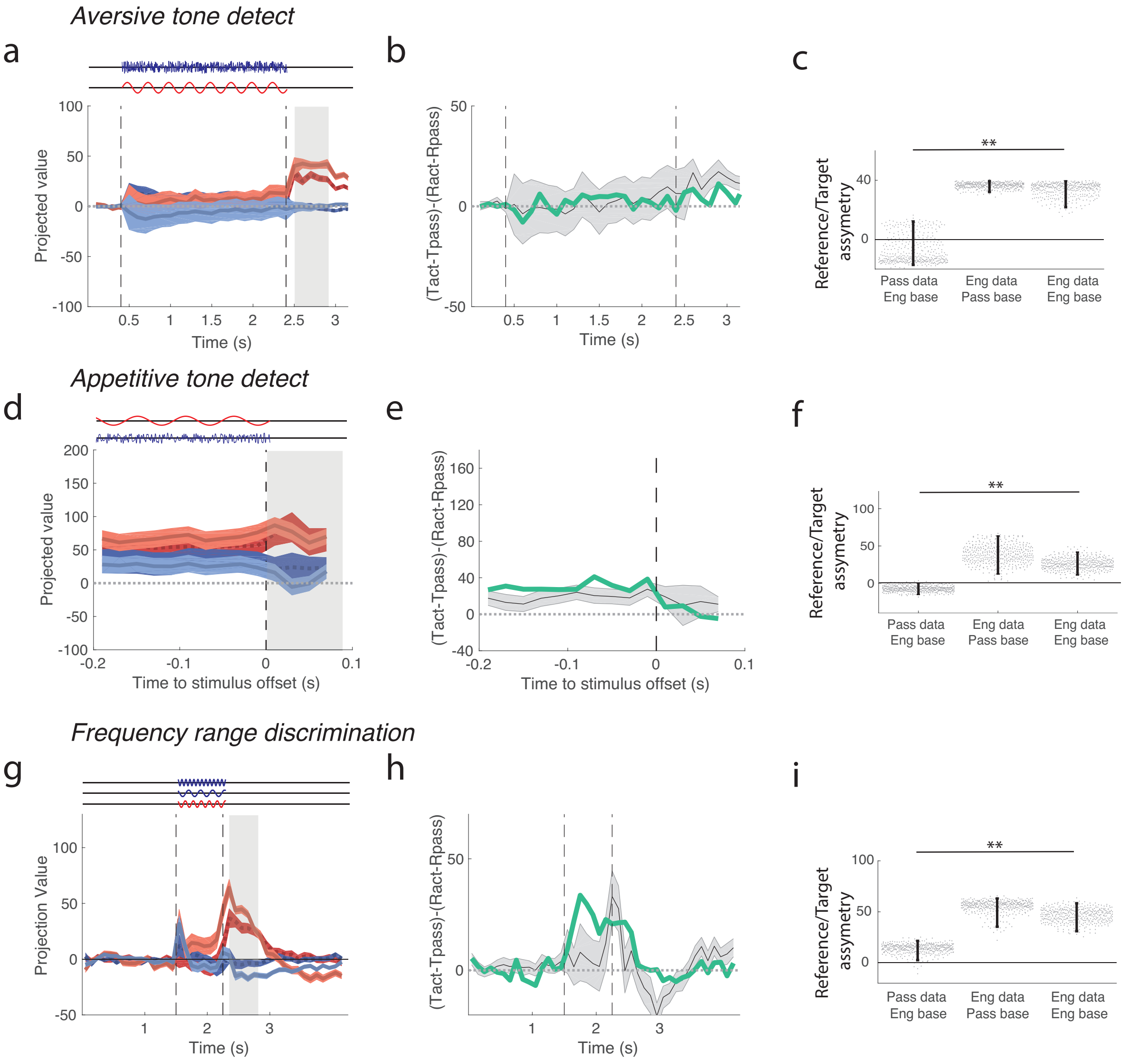

Supplementary Fig 10. Asymmetric encoding of target and reference stimuli in a range of auditory go/no-go tasks during the post-sound silence

a,d,g Projection onto the decoding axis determined during the post-sound silence period of trial-averaged reference (blue) and target (ref) activity during the passive (dark colors) and the active (light colors) sessions. A baseline value computed from pre-stimulus spontaneous activity was subtracted for each neuron, so that the origin corresponds to the projection of spontaneous activity (shown by black line). Note that there is a tendency for the target-driven activity to be further from the baseline in the active state and/or the reference-driven activity to be closer. The periods used to construct the decoding axis are shaded in gray. Error bars represent 1 std calculated using decoding vectors from cross-validation (n=400).

b,e,h Index of target enhancement by task engagement based on projections using the decoding axis determined during post-sound silence. In green same index instead giving the same weight to all units. The difference between the green and black curved indicates that the change in asymmetry induced by task engagement cannot be detected using the population averaged firing rate alone. Error bars represent 1 std calculated using decoding vectors from cross-validation (n=400).

c,f,i Comparison of reference/target asymmetry for evoked responses in different states during the post-sound silence compared to different baselines given by passive or engaged spontaneous

activity. Reference/target asymmetry is the difference of the distance of target and reference projected data to a given baseline. We examine three cases: (i) passive evoked responses, distances calculated relative to engaged spontaneous activity; (ii) engaged evoked responses, distances calculated relative to passive spontaneous activity; (iii) engaged evoked responses, distances calculated relative to engaged spontaneous activity. In all three cases, the engaged decoding axis was used for projections. Error bars represent 95% confidence intervals. ($n=400$ cross validations; Aversive Tone detect: $p(col1,col3)<0.0025$ & $p(col2,col3)=0.92$; Appetitive tone detect; p(col1,col3<0.025 & $p(col2,col3)=0.94$; Frequency range discrimination: $p(col1,col3)<0.0025$ & $p(col2,col3)=0.9$; **: $p<0.01$).

Supplementary Note 1

Population-encoding dynamics change between conditions

In the analyses reported in the main text, we trained a classifier at each time point in the trial, and used it to evaluate stimulus discrimination at the same time point in held-out trials. To assess how much the underlying encoding changes over the trial, we used two procedures. First, we directly compared the classifiers determined at different time-bins by computing the correlation between them (Supplementary Figure 6a,c). Second, we used the classifier obtained at three different trial epochs (early and late stimulus, post-stimulus silence) to classify the neural activity along the whole trials (Supplementary Figure 6b,d). If the encoding of stimulus underlying stimulus discrimination changes over time in the trial, a classifier trained on one time point will lead to a lower discrimination performance at other times.

In the passive condition, we found that changes in encoding over time are weak. The encoding was highly homogeneous within stimulus presentation and during the post-sound silence (Supplementary Figure 6a). Consistent with this view, classifiers trained during the early or the late phases of the stimulus presentation could be used efficiently at all other times during stimulus presentation without an appreciable drop in accuracy (Supplementary Figure 6b, brown and orange curves). In contrast, the same classifier led to chance-level discrimination at time points after stimulus presentation. Conversely a classifier trained after stimulus presentation led to chance-level performance during stimulus presentation (Supplementary Figure 6b, yellow curve). In the passive condition, the neural encoding that underlies stimulus discrimination therefore appears to change very little during stimulus presentation, and shifts abruptly afterwards.

A different picture emerged when animals were engaged in the task. The encoding appeared to change more progressively over the trial (Supplementary Figure 6c), and a classifier trained at one point systematically led to reduced discrimination performance at other time points (Supplementary Figure 6d). Moreover, no sharp transition was apparent at the time the stimulus was switched off. In particular, a classifier trained during the stimulus presentation led to a significant discrimination performance after stimulus presentation (Supplementary Figure 6d, brown and orange curves). Conversely, a classified determined during the post-sound silence led to an above chance and progressively increasing discrimination performance during stimulus presentation (Supplementary Figure 6d, yellow curve).

Altogether, in the engaged condition, the population encoding underlying stimulus discrimination therefore appeared to progressively shift from a representation purely along a stimulus-driven axis, where categorical information was present but uncorrelated with behavior (Fig. 3c top panel), to a representation along a decision-related axis, which was directly correlated with the behavioral action (Fig. 3e bottom panel).

Supplementary Methods

Comparison of results in single and multiunits

All analyses in the main section of the paper concerning the click train discrimination task combine results from single units (isolation distance > 20, see Methods) and multi-units because we found no differences concerning their general properties (see Supplementary Table 1) and the main population-level results of the paper (see Supplementary Table 2) were maintained using SU activity only, although the power of the analysis was of course reduced.

	SU	MU	Comparison
MI : baseline	0.14 +/- 0.03 (***)	0.19 +/- 0.02 (***)	p= 0.22
MI : evoked	0.04 +/- 0.05 (ns)	- 0.05 +/- 0.06 (ns)	p=0.22
MI : vector strength	0.05 +/- 0.006 (***)	0.04 +/- 0.0075 (***)	p=0.25
Ref FR pass. – Snd	7.45 +/- 0.70	6.67 +/- 0.88	p=0.48
Ref FR eng. – Snd	9.14 +/-0.86	8.38 +/- 1.06	p=0.57
Targ FR pass. – Snd	7.78 +/- 0.72	6.15 +/- 0.77	p=0.12
Tar FR eng. – Snd	9.9 +/- 0.93	7.9 +/- 0.97	p=0.15
Ref FR pass. – Sil	6.34 +/-0.64	5.3 +/- 0.68	p=0.25
Ref FR eng. – Sil	7.96 +/-0.76	7.65 +/- 0.94	p=0.79
Targ FR pass. – Sil	6.31 +/-0.67	5.4 +/- 0.76	p=0.36
Targ FR eng – Sil.	8.56 +/-0.84	7.26 +/- 0.99	p=0.32

Supplementary Table 1 Comparison of unit properties for single and multi units. Mean +/- s.e.m are given for each value and the comparison between SU and MU is performed using a ttest. For modulation indexes (first three lines), the significance compared to zero is given in brackets. These results are identical to those found in the main paper.

To verify that the population-level results were maintained SU data, despite the reduced number of units (196 SU units, 370 total units used in main paper), we recapitulate in Supplementary Table 2 the main results using SU activity alone.

	Mean [C.I.] – signif. of comparison
Sound accuracy pass. and eng.	0.97 [0.95:0.99] - 0.98 [0.94:1] **NS**
Silence accuracy pass. and eng.	0.59 [0.52:0.66] - 0.78 [0.69:0.87] *
Sound: ref and target projected values pass.	29 [25:36] - 26 [18:33] **NS**
Silence: ref and target projected values pass.	6 [4:8] - 12 [6:16] **NS**
Sound: ref and target projected values eng.	16 [8:23] - 44 [33:55] **
Silence: ref and target projected values eng.	2 [0.7:4] - 37 [33:42] **

Supplementary Table 2 Recapitulation of important results using SU activity alone.

We found that the significant increase in accuracy during the silence with task engagement was maintained after restriction to SU activity. We also observed the significantly greater role played by target evoked activity in the engaged state after projection (as in Fig. 3) using SU activity alone ($p<0.0025$). The only difference with results given in the main paper is that in the passive state during the silence the stronger contribution of target activity did not achieve significance as in Fig. 3d, bottom.
