# Supplementary material for: Go/No-Go task engagement enhances population representation of target stimuli in primary auditory cortex
Source: Nat Commun. 2018 Jun 28;9:2529. doi: 10.1038/s41467-018-04839-9 (PMC6023878; doi:10.1038/s41467-018-04839-9)
Supplement: Supplementary file 2 — Peer Review File [file 41467_2018_4839_MOESM2_ESM.pdf]

Editorial Note: This manuscript has been previously reviewed at another journal that is not operating a transparent peer review scheme. This document only contains reviewer comments and rebuttal letters for versions considered at Nature Communications.
